# Holistic Approaches to Arrhythmia Management: Combining Medication, Ablation, and Device Interventions

**DOI:** 10.7759/cureus.45958

**Published:** 2023-09-25

**Authors:** Mitul Hareshkumar Chaudhary, Shah Dev, Ankeeta Kumari, Kainat Kanwal, Dhruvkumar N Jadav, Sohaib Rasool, Muhammad Tayyab Saleem, Ridhi Bhagat, FNU Prachi, Piyush Puri, Maham Kashif, Giustino Varrassi, Mahima Khatri, Satesh Kumar, Tamam Mohamad

**Affiliations:** 1 Medicine, AMA School of Medicine, Makati, PHL; 2 Medicine, Jinnah Sindh Medical University, Karachi, PAK; 3 Medicine, Liaquat University of Medical and Health Sciences, Jamshoro, PAK; 4 Medicine, Khawaja Muhammad Safdar Medical College, Sialkot, PAK; 5 Medicine, Pandit Deendayal Upadhyay Medical College, Rajkot, IND; 6 Medicine, Bakhtawar Amin Medical and Dental College, Multan, PAK; 7 Medicine, Services Institute of Medical Sciences, Lahore, PAK; 8 Internal Medicine, Teerthanker Mahaveer Medical College and Reseach Center, Moradabad , IND; 9 Medicine, Guru Teg Bahadur Hospital, Delhi, IND; 10 Internal Medicine, Adesh Institute of Medical Science and Research, Bathinda, IND; 11 Pain Medicine, Paolo Procacci Foundation, Rome, ITA; 12 Medicine and Surgery, Dow University of Health Sciences, Karachi, PAK; 13 Medicine and Surgery, Shaheed Mohtarma Benazir Bhutto Medical College, Karachi, PAK; 14 Cardiovascular Medicine, Wayne State University, Detroit, USA

**Keywords:** comprehensive care, interventions, ablation device, medication catheter, holistic approaches, arrhythmia management

## Abstract

This narrative review investigates the severe health issue of arrhythmias, which affects millions of people worldwide. A multifaceted strategy incorporating medicine, catheter ablation, and advanced device interventions is necessary to manage these disorders effectively. Medication is the cornerstone, and as antiarrhythmic medications develop, their efficacy and side effects are reduced. Success depends on having individualized treatment strategies that consider patient profiles and arrhythmia type. Catheter ablation, a minimally invasive surgery that targets and removes faulty heart electrical circuits, has become a potent therapy when drugs are ineffective. Technological developments, including high-resolution mapping systems and customized catheters, improve precision. Pacemakers and implantable cardioverter-defibrillators (ICDs) are two examples of implantable cardiac devices essential to managing all types of arrhythmias. Pacemakers provide a regular heartbeat when the body's natural pacing mechanism fails. At the same time, ICDs, with cutting-edge algorithms, can identify and stop life-threatening arrhythmias and offer high-risk patients vital protection. As device technology advances, smaller, more durable devices become available, improving patient comfort and lowering the need for replacements. The seamless fusion of these three strategies is where holistic arrhythmia management shines. Even for difficult instances, customized combination therapy combining medicine, ablation, and device interventions offers complete solutions. Healthcare providers must collaborate for this integrated strategy to deliver personalized, efficient, and holistic care. In conclusion, the management of arrhythmias has developed into a dynamic, synergistic discipline where drugs, catheter ablation, and devices all work in concert to deliver comprehensive care. For those with arrhythmias, a patient-centered strategy that considers their particular patient features and best integrates different modalities can significantly enhance their quality of life. The effectiveness and accessibility of holistic arrhythmia management could be further improved because of ongoing developments in these fields, which is encouraging for patients and medical professionals.

## Introduction and background

Millions of people worldwide are impacted by arrhythmias, which are widespread and clinically severe cardiovascular challenges. Abnormal heart rhythms define arrhythmias. These disturbances in the heart's usual electrical activity can cause palpitations, an irregular heartbeat, light-headedness, vertigo, or, in more severe situations, potentially fatal complications, including a stroke or sudden cardiac death. They cover a wide range of heart rhythm problems, such as atrial fibrillation (AF), which affects the upper chambers of the heart, bradycardia (slow heart rhythms), and tachycardia (rapid heart rhythms). Arrhythmias are prevalent and rising, especially as the world's population ages. One of the most prevalent types of arrhythmias is AF, which has drawn much attention due to its frequency and potential hazards. Approximately 2.7 to 6.1 million people in the United States alone suffer from AF, and by 2030, there will likely be 12.1 million instances worldwide [1].

This illness seriously threatens the public's health because it increases the risk of stroke by five times. Additionally, ventricular arrhythmias, such as ventricular tachycardia (VT) and ventricular fibrillation (VF), can result in sudden cardiac death, accounting for more than 300,000 fatalities annually in the United States [2]. Aside from causing death, arrhythmias significantly negatively influences patients' quality of life and place a heavy financial burden on healthcare systems. The cost of overall healthcare for these disorders is influenced by hospitalizations, diagnostic tests, treatments, and long-term management related to arrhythmias [3]. Therefore, there is an urgent need to efficiently and thoroughly manage arrhythmias. Historically, pharmacological medications have been employed mainly for symptom alleviation and rhythm control in managing arrhythmias. While drugs remain a crucial component of arrhythmia care, they sometimes don't offer long-term fixes and may only be partially effective in treating some arrhythmias [3,4]. Additionally, they might not deal with the root causes of arrhythmias or lessen the possible effects. In recent years, a considerable trend toward holistic methods of managing arrhythmias has occurred. Arrhythmias are not isolated events; holistic therapy understands that they frequently originate from underlying heart illnesses, anatomical abnormalities, or systemic causes [5]. As a result, it stresses combining many therapy methods to address the underlying causes and potential side effects of arrhythmias and control the rhythm.

This narrative review's goal is to thoroughly examine all available holistic care options for arrhythmias, focusing on the integration of medicine, catheter ablation, and cutting-edge technology interventions. The function of pharmaceuticals in managing arrhythmias will be examined in detail, along with their development, effectiveness, and significance in individualized treatment strategies catered to patient profiles that may include age, comorbidities, and particular arrhythmias. We will go into great detail on catheter ablation, a minimally invasive technique used to target and remove faulty electrical circuits in the heart. The review will examine its success rates, indications, technological developments in ablation (such as high-resolution mapping systems and contact force sensor catheters), and circumstances where ablation might be chosen for medicine. Exploring device interventions is integral to a comprehensive approach in managing arrhythmias, with pacemakers and implantable cardioverter-defibrillators (ICDs), among other cardiac implantable devices, playing vital roles in this strategy. The functions of these devices, their contributions to preserving normal heart rhythms, and technological improvements in the form of gadget shrinking and longer battery life will all be covered in this review.

The review will stress the significance of smoothly combining medicine, catheter ablation, and device therapies to provide complete treatment for patients with arrhythmias. It will review the advantages of such an integrated strategy and customized combination therapies that treat difficult arrhythmia conditions. Collaboration amongst medical experts, including cardiologists, electrophysiologists, nurses, and other allied healthcare providers, will be essential for providing comprehensive, tailored care for managing arrhythmias. This study intends to advance knowledge of the subject and offer insights into the changing landscape of arrhythmia treatment by critically analyzing various facets of holistic care. It will also emphasize how continuous improvements in drugs, ablation methods, and device technology can further improve the efficiency and accessibility of holistic arrhythmia therapy, ultimately bringing hope and better outcomes for patients and physicians.

## Review

Methodology

Goals and the Research Question

This narrative review's main goal was to thoroughly examine and integrate the knowledge on holistic approaches to the therapy of arrhythmias, emphasizing the integration of medication, catheter ablation, and sophisticated technology therapies. What is the present level of knowledge in this field and how do holistic approaches that incorporate medication, catheter ablation, and device interventions help manage arrhythmias? This was the overall research question that served as the basis for this review.

Search Techniques

To find pertinent studies and literature on arrhythmia management, a thorough search was carried out in credible electronic databases, including PubMed, MEDLINE, Embase, Scopus, and the Cochrane Library. Keywords and controlled vocabulary terms (MeSH phrases) were combined for the search. The phrases "arrhythmia management," "antiarrhythmic drugs," "catheter ablation," and "implantable cardiac devices" and their derivatives were among them. The search strategy was created with inclusivity and topical relevancy in mind.

Inclusion Criteria

Studies from the 2013-2023 period that have been published in English; papers based on original research, comprehensive reviews, meta-analyses, clinical recommendations, and consensus statements; studies specifically focused on integrating medicine, catheter ablation, and device therapies to manage arrhythmias holistically-investigations involving people

Exclusion Criteria

Studies that are unrelated to the subject of comprehensive arrhythmia management; studies with poor methodology or too little connection to the goals of the review; pediatric populations, animals, or non-human individuals were the main subjects of studies; publications in languages besides English

Selection of Studies

Screening: A preliminary review of article titles and abstracts was done to find any that might be pertinent-removing duplicate records and blatantly irrelevant articles.

Review of the full text: Potentially pertinent studies' full texts were retrieved, and their eligibility was evaluated. Each paper was assessed for inclusion by two independent reviewers, and disagreements were settled through discussion or, if necessary, contact with a third reviewer.

Extraction of Data

Data were methodically extracted from several studies. The study's design, the year it was published, patient characteristics, the interventions (medication, ablation, devices), the results, and the conclusions on integrating these all-encompassing techniques were vital data items. A structured table was used to record the data for simple access.

Analyzing and Synthesizing Data

The narrative review included a qualitative synthesis of the information from the chosen studies' conclusions. The information's organization and narrative presentation made a thorough overview of the subject possible.

Quality Evaluation

The caliber of the included studies was evaluated using pertinent techniques, such as the Joanna Briggs Institute (JBI) critical assessment checklist for different study categories. This assessment assisted in determining the validity and dependability of the reviewed data.

Ethics-Related Matters

Ethics approval was unnecessary for this narrative review because it combined previously published literature. However, moral standards were scrupulously upheld, including correct citation and observance of copyright laws.

Reporting

The narrative review's conclusions were presented in a clear, organized manner. The review followed accepted reporting standards for narrative reviews and comprised sections on introduction, methods, results, discussion, and conclusion. This methodology describes the systematic, in-depth narrative review of "Holistic Approaches to Arrhythmia Management: Combining Medication, Ablation, and Device Interventions." To provide insightful information on this crucial aspect of arrhythmia treatment, it ensured that pertinent studies were included and the data was synthesized.

Arrhythmias: Understanding the challenge

A set of heart rhythm diseases known as arrhythmias, often referred to as cardiac arrhythmias, is characterized by irregular heartbeats. The word "arrhythmia" denotes a change from the heart's typical rhythm. The heart usually beats in a regular, coordinated pattern to properly pump blood throughout the body. However, when this rhythm is thrown off, it can result in several clinical symptoms and harm one's health. This essay describes arrhythmias, and their classification clarifies how they affects patients clinically, and highlights the threat they bring to global health.

Definition and Classification of Arrhythmias

Arrhythmias are characterized as deviations from normal cardiac depolarization and repolarization in terms of frequency, rhythm, or sequence, which can lead to irregular heartbeats. These deviations may appear as erratic heartbeats, too rapid (tachycardia), sluggish (bradycardia), or even wholly erratic rhythms. Arrhythmias can develop in the atria, ventricles, or specific conduction routes, among other areas of the heart [5]. Arrhythmias must be divided into numerous types to be better understood.

Bradycardia: This type of arrhythmia is distinguished by a slow heartbeat, typically less than 60 beats per minute. It may result from problems with the sinoatrial (SA) node, the conduction pathways, or the heart's natural pacemaker.

Tachycardia: Contrarily, tachycardia is characterized by an unusually high heart rate that frequently exceeds 100 beats per minute. This can happen for several reasons, such as ventricular or atrial anomalies.

AF: One of the most prevalent arrhythmias, AF is characterized by erratic and fast atrioventricular (AV) contractions. It raises the danger of stroke and other heart-related issues.

VF: VF is a potentially fatal arrhythmia in which the ventricles quiver instead of contracting. If untreated, VF can result in a lack of blood supply and rapid cardiac arrest.

Supraventricular tachycardia (SVT): This is a tachycardia that starts in the atria or the AV node, where it frequently originates. The Wolff-Parkinson-White syndrome (WPW) is a congenital arrhythmia that can cause problems and rapid heartbeats by creating an aberrant auxiliary electrical pathway between the atria and ventricles.

Arrhythmias' Clinical Effects on Patients

Arrhythmias can have a significant clinical impact on patients, ranging from minor symptoms to potentially fatal situations. They can cause various symptoms, such as palpitations, fainting spells, dizziness, weariness, and chest pain. They can occasionally be asymptomatic and can only be identified through regular medical exams or electrocardiogram (ECG) testing. The increased risk of stroke is one of the most severe consequences connected to arrhythmias [6]. Particularly with AF, blood can accumulate in the atria, forming clots that can go to the brain and result in a stroke. According to estimates, AF more than doubles the risk of stroke. A sudden cardiac arrest can also result from persistent ventricular arrhythmias such as VT or VF. Without prompt treatment (usually defibrillation), sudden cardiac arrest, a life-threatening condition in which the heart abruptly stops functioning, can cause death within minutes.

Arrhythmias have an influence that goes beyond short-term health issues. Due to the unpredictable nature of their symptoms, potential restrictions on their ability to engage in certain activities, and the emotional strain brought on by chronic heart illness, patients with arrhythmias frequently feel a lower quality of life. The cost of treating arrhythmias and the complications they cause, such as hospital stays and medicines, is also substantial. Due to their incidence and related morbidity and mortality, arrhythmias pose a significant threat to world health. The World Health Organization (WHO) reports that arrhythmias and other cardiovascular illnesses are the leading causes of death globally, accounting for over 17.9 million fatalities annually [7]. Arrhythmias significantly contribute to these deaths, especially sudden cardiac deaths. Several causes will likely cause the worldwide burden of arrhythmias to increase in the upcoming years. First, arrhythmias are more common in older people and are predicted to become more common as life expectancy rises [8]. The second reason contributing to the development of arrhythmias is the rising incidence of risk factors such as obesity, diabetes, and hypertension [9]. Third, more patients are receiving diagnoses for arrhythmias due to greater access to healthcare and diagnostic tools [10]. Arrhythmias pose a global problem that needs to be addressed from multiple angles. Preventive measures include promoting healthy eating habits, frequent exercise, and quitting smoking to lower the risk factors for arrhythmias [11].

Early Detection and Diagnosis

To detect arrhythmias early, it is recommended to encourage routine cardiovascular screenings and ECG monitoring, especially in high-risk populations [12].

Treatment and management: Providing access to the necessary therapies, such as prescription drugs, cardiac devices (such as pacemakers and ICDs), and catheter-based techniques like ablation therapy [13].

Public awareness and education: Informing people about the symptoms and signs of arrhythmias and the value of receiving prompt medical assistance [14].

Research and innovation: Encourage the development of new treatments and research projects to better understand the causes underlying arrhythmias [15]. As a whole, arrhythmias constitute a wide range of cardiac rhythm problems with severe clinical repercussions for patients, such as an elevated risk of stroke, abrupt cardiac arrest, and a reduced quality of life. Arrhythmias significantly threaten world health by significantly increasing the burden of illness and mortality. The solution to this problem involves a multifaceted strategy that includes public awareness, prevention, early identification, efficient treatment, and ongoing research and innovation.

Medication in arrhythmia management

Arrhythmias, also known as cardiac rhythm disorders, comprise a broad spectrum of irregular heartbeats that pose grave health risks. Effective management of arrhythmias is essential for enhancing the quality of life of affected individuals and reducing associated morbidity and mortality. In treating arrhythmias, medication serves as the cornerstone of therapy for many patients [15]. This essay will examine the significance of medication as the primary approach to arrhythmia management, the evolution of antiarrhythmic drugs and their efficacy, and the significance of individualized treatment plans based on patient profiles.

Medication's Crucial Function in Arrhythmia Treatment

Medication plays a crucial role in the management of arrhythmias, in many instances serving as the primary intervention. The goals of antiarrhythmic drugs are to restore and maintain a normal cardiac rhythm, prevent arrhythmia recurrence, alleviate symptoms, and reduce the risk of complications such as stroke and heart failure [15]. Depending on their specific mechanisms of action, medications can be broadly classified as rhythm-control or rate-control substances. These medications seek to restore and maintain a normal sinus rhythm by suppressing or eliminating abnormal electrical impulses in the heart. They are particularly beneficial for patients with AF or atrial flutter, where the aim is to convert the irregular rhythm into a regular one. Common medications for rhythm control include amiodarone, flecainide, and propafenone [16]. Rather than restoring a normal heart rhythm, rate-control medications seek to slow the heart rate in patients with arrhythmias such as AF. Patients who do not respond well to rhythm-control strategies or are at risk for adverse effects from antiarrhythmic medications may benefit from this method. Commonly used for rate control are beta-blockers (e.g., metoprolol) and calcium channel blockers (e.g., verapamil) [16]. Even though antiarrhythmic drugs can be highly effective in managing arrhythmias, their use is not challenging. Some medications can cause proarrhythmic effects, which may exacerbate or induce arrhythmias. In addition, the choice of medication and dosage must be tailored to the specific form of arrhythmia and the patient's overall health, highlighting the importance of individualized treatment plans.

The Development of Antiarrhythmic Drugs and Their Effectiveness

Antiarrhythmic drug development and evolution have been a dynamic process driven by advances in our knowledge of cardiac electrophysiology and drug safety. Both successes and failures have marked the development of antiarrhythmic medications.

Class I antiarrhythmics: These medications primarily inhibit sodium channels in cardiac cells. They were divided into subclasses according to their effects on sodium channels (e.g., Classes IA, IB, and IC). However, their proarrhythmic potential and limited efficacy in certain cases necessitated their use with caution. For instance, while procainamide (Class IA) and lidocaine (Class IB) were widely employed, they each had specific limitations [17].

Class II antiarrhythmics: Beta-blockers, which are classified as Class II antiarrhythmics, have proven to be highly effective in the management of arrhythmias, particularly in preventing episodes of AF and VT. These medications decrease heart rate and myocardial oxygen consumption, benefiting both rate and rhythm control [16].

Class III antiarrhythmics: Agents of Class III, such as amiodarone and sotalol, are now among the most commonly used antiarrhythmics. Particularly, amiodarone has demonstrated efficacy in treating various arrhythmias, including atrial and ventricular arrhythmias, although its long-term use may be limited by the risk of adverse effects [17].

Class IV antiarrhythmics: Calcium channel blockers, classified as Class IV antiarrhythmics, are primarily used for pulse control. For AF and other tachyarrhythmias, verapamil and diltiazem are frequently prescribed [17].

The search has marked the development of antiarrhythmic pharmaceuticals for agents with greater efficacy and fewer side effects. However, the proarrhythmic potential of certain drugs and the difficulty of selecting the most suitable drug for individual patients highlights the need for a personalized approach to arrhythmia treatment [16].

Importance of Individualized Treatment Plans Determined by Patient Profiles

One of the most challenging aspects of arrhythmia management is tailoring treatment to each patient's specific requirements and characteristics. Creating a personalized treatment plan requires considering factors such as the type of arrhythmia, the patient's overall health, comorbidities, and drug tolerance [18]. Evaluation of risk is essential for determining the most effective treatment strategy. Patients with AF, for instance, must be evaluated for their risk of stroke using instruments such as the CHA2DS2-VASc score. Those at higher risk may require anticoagulation therapy and antiarrhythmic medications [18]. Patients with arrhythmias frequently have other medical conditions requiring cautious management, such as heart failure or hypertension. The choice of antiarrhythmic pharmaceuticals should take into account potential drug interactions and the impact of comorbidities [18]. Individuals respond differently to antiarrhythmic pharmaceuticals in terms of efficacy and tolerability. Some patients may experience adverse effects or insufficient symptom control with a specific medication, necessitating adjustments or alternative options. Regular monitoring of patients receiving antiarrhythmic therapy is essential for determining the drug's efficacy and safety. This includes ECG monitoring, assessment of blood drug levels (e.g., for medications such as digoxin), and evaluation of renal and hepatic function, as many antiarrhythmics are metabolized by these organs [19]. Critical is the collaborative, informed decision-making between patients and healthcare professionals. Patients should be educated on the risks and benefits of treatment options and potential adverse effects and actively participate in decision-making. Recent advances in genetic and molecular medicine have enabled a more individualized approach to managing arrhythmia. Individuals with specific genetic mutations predisposing them to certain arrhythmias can be identified through genetic testing, thereby guiding treatment decisions. In addition, novel therapies such as catheter ablation, which targets the specific regions of the heart responsible for arrhythmias, offer a tailored, minimally invasive treatment approach. 

Catheter ablation: A powerful intervention

Catheter ablation is a highly effective and widely employed therapeutic intervention in cardiac electrophysiology, used predominantly for managing cardiac arrhythmias. The evolution of this procedure over the past several decades has significantly enhanced patient outcomes. In this in-depth analysis, we will define catheter ablation and its role in arrhythmia control, describe the procedure and its targeting of abnormal electrical pathways, discuss success rates and circumstances where ablation is preferred over medication, and present recent advancements in ablation technology, including mapping systems and catheter types.

Catheter Ablation and Its Role in Arrhythmia Management

Catheter ablation is a minimally invasive procedure used to treat cardiac arrhythmias, which are abnormal heart rhythms that can contribute to stroke, heart failure, and even sudden cardiac death. Identifying and obliterating the source or pathway of aberrant electrical impulses within the heart is the fundamental principle underlying catheter ablation. This is accomplished by inserting specialized catheters into the heart through blood vessels, typically the femoral vein, and guiding them to the target site with fluoroscopy and electroanatomic mapping systems [20].

Method and Identification of Abnormal Electrical Pathways

Utilizing radiofrequency (RF) energy or cryotherapy, the aberrant tissue responsible for the arrhythmia is eliminated or isolated. Electroanatomic mapping systems, such as CARTO or EnSite, provide real-time three-dimensional heart mapping, allowing precise localization of arrhythmogenic areas [21]. Once the abnormal site has been identified, the catheter is positioned in direct contact with the tissue, and energy is applied to generate controlled lesions, thereby interrupting the abnormal electrical pathway and restoring normal rhythm. The success of catheter ablation depends on precise tissue mapping, deft catheter manipulation, and comprehensive tissue ablation.

Rates of Success and Preferences Regarding Medication

Ablation catheters have demonstrated remarkable success rates in treating a variety of arrhythmias. In the case of AF, one of the most prevalent arrhythmias, catheter ablation has demonstrated long-term success rates of up to 70-80% in attaining freedom from recurrent AF episodes [22]. In contrast, antiarrhythmic drugs, commonly used to control arrhythmias, are frequently ineffective and can cause adverse effects. A variety of factors influence the preference for catheter ablation over medication. First, ablation offers the possibility of a definitive remedy, whereas medications typically treat symptoms without addressing the underlying cause. Patients who do not respond well to medications or who experience intolerable adverse effects may choose ablation instead. In addition, certain arrhythmias, such as atrial flutter and SVT, have a long history of successful ablation outcomes, making it the preferred treatment modality [20].

Recent Technological Advancements in Ablation

Recent technological advances in catheter ablation have enhanced its efficacy and safety. Notably, electroanatomic mapping systems have become more sophisticated, enabling faster and more precise mapping of the cardiac chambers. Utilizing high-density mapping catheters, such as the Advisor HD Grid mapping catheter, improves spatial resolution and can shorten procedure times [23]. The development of contact force-sensing catheters is another significant development. These catheters measure the force applied to the tissue during ablation, ensuring uniform lesion formation and minimizing the risk of complications [24]. In addition, technological advances in catheter design have led to the creation of catheters with advanced cooling systems, such as irrigated-tip catheters. These catheters enable more controlled and efficient RF energy delivery, reducing the risk of complications such as steam pops and perforations [25]. In addition to technological advancements, research is ongoing into alternative energy sources, such as laser ablation and ultrasound, which may provide additional ablation therapy options in the future [26]. Catheter ablation has firmly established itself as a potent technique for managing cardiac arrhythmias. Targeting aberrant electrical pathways within the heart offers a potential cure and better outcomes than medical therapy. Recent advances in ablation technology, such as mapping systems and catheter designs, have further enhanced the procedure's safety and efficacy. Catheter ablation is likely to play an increasingly important role in the management of arrhythmias, offering patients a path to a healthier and more rhythmically stable existence as research progresses.

Device interventions in holistic arrhythmia management

The management of arrhythmias has been revolutionized by implantable cardiac devices, such as pacemakers and ICDs. These devices play a crucial role in maintaining a normal heart rate, detecting and terminating life-threatening arrhythmias, and enhancing the overall quality of life for people with arrhythmia-related conditions. In this in-depth discussion, we will introduce implantable cardiac devices, delve into the roles of pacemakers and ICDs, and examine the most recent technological advancements, such as miniaturization and enhanced battery life.

Pacemakers and Implantable Cardiovascular Devices

Implantable cardiac devices are small electronic devices surgically implanted in the thorax to treat heart rhythm disorders. ICDs and pacemakers are the two most common implantable devices. Pacemakers are devices that treat bradycardia, characterized by an abnormally sluggish heart rate. They consist of an implanted pulse generator and one or more leads (wires). When the heart's natural electrical conduction system is not functioning correctly, the pulse generator stimulates the heart muscle to contract by delivering electrical impulses [26]. ICDs are sophisticated devices used to treat life-threatening ventricular arrhythmias like VT and VF. ICDs, like pacemakers, are composed of a pulse generator and leads. Nevertheless, ICDs feature sophisticated sensing and defibrillation capabilities. They can detect potentially fatal abnormal heart rhythms and deliver electrical impulses to restore normal rhythm, thereby preventing cardiac arrest [27].

Pacemaker Functions in Maintaining a Regular Heart Rate

Pacemakers are instrumental in sustaining a regular heart rate, particularly in patients with bradycardia or heart block. The pacemaker takes over when the SA node, the heart's natural pacemaker, fails to initiate electrical impulses or when there is a blockage in the conduction pathways. Pacemakers are programmable devices that enable clinicians to tailor their functionality to each patient's specific needs. They can detect the heart's intrinsic electrical activity and deliver the necessary electrical impulses. This ensures that the heart rate is adequate to meet the oxygen and metabolic demands of the body. Pacemakers also alleviate symptoms of bradycardia, such as fatigue, vertigo, and fainting, thereby improving the patient's quality of life [28].

ICDs: Detecting and Terminating Dangerous Arrhythmias

ICDs are essential to arrhythmia management, particularly for those at risk of life-threatening ventricular arrhythmias. These devices have various detection algorithms that distinguish between normal and abnormal heart rhythms and perpetually monitor the heart's rhythm. When an ICD detects a ventricular arrhythmia, it can administer therapies to terminate it. Anti-tachycardia pacing, which administers rapid pacing to interrupt the arrhythmia or high-energy shocks, can restore a normal rhythm. ICDs respond rapidly, typically within milliseconds, to prevent sudden cardiac death induced by ventricular arrhythmias [29].

Innovations in Device Technology

Recent technological advancements in implantable cardiac devices have improved performance and patient outcomes. The miniaturization of device components is a significant technological advance. Smaller devices reduce the size of the surgical site, minimize disfigurement, and lessen the likelihood of complications. In addition to being more comfortable for patients, these miniaturized devices can be implanted using minimally invasive procedures [30]. Additionally, battery technology has advanced, resulting in extended device life spans. Longer battery life decreases the frequency of device replacement operations, improving patient convenience and lowering healthcare costs [31]. Modern implantable medical devices are equipped with remote monitoring capabilities. This allows healthcare providers to continuously monitor device function and patient status, facilitating early detection of issues and timely intervention. It has been demonstrated that remote monitoring reduces hospitalizations and improves patient care [32].

Leadless pacemakers are a revolutionary advancement. Unlike traditional pacemakers with leads, these devices are entirely within the heart. Leadless pacemakers are less invasive, carry a lower risk of lead-related complications, and have cosmetic appeal [33]. Some newer implantable devices are MRI-compatible, allowing patients to endure necessary diagnostic imaging without the risks associated with older devices [33]. Implantable cardiac devices, such as pacemakers and ICDs, have revolutionized arrhythmia management. These devices make it possible to maintain regular heart rhythms, prevent sudden cardiac death, and improve the quality of life for people with arrhythmia-related conditions. Advances in device technology, such as miniaturization, longer battery life, and remote monitoring, continue to enhance patient outcomes and the overall efficacy of arrhythmia management strategies. These advancements highlight the significance of device interventions in comprehensive arrhythmia management, offering patients a more promising and secure cardiac future.

The synergy of holistic arrhythmia management

Arrhythmias, characterized by abnormal heart rhythms, pose a global health concern. The management of arrhythmias encompasses a multidisciplinary approach that includes medication, catheter ablation, and device interventions. Integrating these modalities into a comprehensive treatment plan maximizes the chances of success and provides individualized care for patients. This discussion will elaborate on how medication, catheter ablation, and device interventions can be effectively integrated, the benefits of combining these approaches, the crucial role of individualized treatment plans, and examples of successful holistic arrhythmia management cases.

Integration of Medication, Catheter Ablation, and Device Interventions

Medication: Medications, known as antiarrhythmics, are often the initial approach in managing arrhythmias. They work by stabilizing the heart's electrical activity, reducing abnormal rhythms, and alleviating symptoms. These medications are tailored to the specific arrhythmia type and the patient's overall health. Medications can be used as standalone therapy or in conjunction with other interventions [34].

Catheter ablation: Catheter ablation is a minimally invasive procedure that targets and eliminates the abnormal electrical pathways causing arrhythmias. During ablation, specialized catheters are guided to the affected area of the heart, and either RF energy or cryotherapy is delivered to create controlled lesions, interrupting the faulty electrical circuits. Ablation can offer a curative approach for certain arrhythmias, reducing or eliminating the need for long-term medication [20].

Device interventions: Implantable devices, such as pacemakers and ICDs, are pivotal in arrhythmia management. Pacemakers maintain a regular heart rate for individuals with bradycardia, whereas ICDs detect and terminate life-threatening arrhythmias, such as VT or VF, preventing sudden cardiac death [26].

Benefits of Combining Approaches for Comprehensive Care

The integration of medication, catheter ablation, and device interventions offers several compelling benefits:

Maximized efficacy: Combining therapies allows clinicians to target arrhythmias from multiple angles, increasing the likelihood of success. Medication can help control symptoms while awaiting or following ablation, and devices can provide a safety net for individuals at risk of life-threatening arrhythmias [35].

Reduced medication burden: Catheter ablation can provide a curative solution for certain arrhythmias, allowing patients to reduce or discontinue antiarrhythmic medications. This can improve the patient's overall quality of life and reduce the risk of medication-related side effects [36].

Individualized treatment: Integrating these approaches allows for highly individualized care. The choice of therapy depends on the patient's specific arrhythmia type, overall health, lifestyle, and preferences. This personalized approach ensures patients receive the most suitable treatment for their unique circumstances [37].

Long-term success: Catheter ablation, when successful, can offer long-term freedom from arrhythmias. This reduces the need for ongoing medication management and provides patients peace of mind [38].

The Importance of Individualized Treatment Plans

Holistic arrhythmia management emphasizes the significance of individualized treatment plans tailored to each patient's needs and preferences. This approach includes several key elements:

Comprehensive assessment: A thorough evaluation is essential, considering the patient's medical history, symptoms, diagnostic tests, and underlying heart condition. This assessment helps in accurate diagnosis and treatment planning.

Shared decision-making: Engaging patients in shared decision-making empowers them to participate in their care actively. Clinicians should discuss treatment options, potential risks and benefits, and align interventions with patients' values and preferences.

Multidisciplinary collaboration: Effective arrhythmia management often involves a multidisciplinary team of healthcare professionals, including cardiologists, electrophysiologists, cardiac surgeons, and nurses. This collaborative approach ensures a comprehensive evaluation and a wide range of treatment options [37].

Long-term follow-up: Continuous monitoring and follow-up are critical to assess treatment outcomes, adjust medication regimens, and ensure the ongoing effectiveness of device interventions. Regular check-ups help detect issues early and allow timely intervention [30].

Examples of Successful Holistic Arrhythmia Management Cases

Case 1: AF-A 55-year-old individual with persistent AF experiencing palpitations and fatigue. The patient was started on antiarrhythmic medication to control AF symptoms while awaiting catheter ablation. Ablation successfully restored normal sinus rhythm. The patient remained arrhythmia-free after ablation, allowing for discontinuation of antiarrhythmic medication and significantly improved quality of life [22].

Case 2: V-A 40-year-old with a history of myocardial infarction and recurrent VT episodes. The patient underwent catheter ablation targeting the VT origin. Additionally, an ICD was implanted for secondary prevention. Catheter ablation significantly reduced VT episodes, and the ICD successfully terminated life-threatening VT episodes, preventing sudden cardiac death [30].

Case 3: Bradycardia-A 65-year-old with symptomatic bradycardia and frequent dizziness. The patient received a dual-chamber pacemaker to maintain a regular heart rate. With the pacemaker in place, the patient experienced a substantial improvement in heart rate, eliminating symptoms and enhancing overall quality of life [26]. These cases underscore the synergy of holistic arrhythmia management, where medication, catheter ablation, and device interventions were tailored to each patient's specific needs, resulting in successful outcomes and improved quality of life. Holistic arrhythmia management involves the integration of medication, catheter ablation, and device interventions to provide comprehensive care for patients with arrhythmias. This approach maximizes efficacy, reduces medication burden, and offers highly individualized treatment plans. Successful cases highlight the potential for improved quality of life and enhanced outcomes through holistic arrhythmia management. Clinicians can optimize patient outcomes and improve overall well-being by emphasizing individualized care and leveraging the strengths of various treatment modalities.

Collaborative healthcare approach

Healthcare is a complex and multidimensional field, and nowhere is collaboration more critical than in the care of patients with cardiovascular conditions, including arrhythmias. The management of arrhythmias requires a multidisciplinary team approach that includes cardiologists, electrophysiologists, and other healthcare professionals. This approach ensures individualized, effective, and holistic care for patients. In this discussion, we will highlight the need for collaboration among these specialists and explain how a multidisciplinary team ensures comprehensive care.

The Need for Collaboration

Diverse expertise: Cardiovascular conditions, including arrhythmias, encompass a wide range of complexities. Cardiologists and electrophysiologists bring different areas of expertise to the table. Cardiologists are trained in diagnosing and managing cardiovascular diseases broadly, while electrophysiologists specialize in the electrical properties of the heart and the diagnosis and treatment of arrhythmias. Collaboration between these specialists ensures a more comprehensive understanding and management of patients' conditions [20].

Comprehensive assessment: Arrhythmia patients often have multiple comorbidities and complex medical histories. Collaboration among healthcare professionals allows for a comprehensive assessment of the patient's overall health. This can include evaluating the impact of arrhythmias on other organs and systems, such as the kidneys and lungs, to provide more tailored treatment plans [23].

Innovations in treatment: Arrhythmia management is a rapidly evolving field, with new treatment modalities and technologies constantly emerging. A collaborative approach facilitates the exchange of knowledge and expertise, ensuring that patients have access to the latest advancements in care [24].

Components of a Multidisciplinary Team

A multidisciplinary team involved in arrhythmia management typically includes:

Cardiologists: General cardiologists play a fundamental role in diagnosing arrhythmias and managing patients' overall cardiovascular health. They often refer patients to electrophysiologists for specialized arrhythmia care.

Electrophysiologists: These are cardiologists with specialized training in the electrical properties of the heart. They perform complex procedures like catheter ablation and implantation of cardiac devices.

Cardiac surgeons: In some cases, especially for arrhythmias that require surgical intervention, cardiac surgeons may be part of the team. They can perform procedures like the Maze procedure for AF.

Nurses and nurse practitioners: Nurses play a crucial role in patient education, pre- and post-procedure care, and monitoring. Nurse practitioners can also assist in managing arrhythmia patients, particularly in outpatient settings [39].

Radiologists and imaging specialists: Imaging plays a significant role in diagnosing arrhythmias and guiding interventions. Radiologists and imaging specialists assist in interpreting diagnostic tests like echocardiograms, MRIs, and CT scans.

Anesthesiologists: For procedures like catheter ablation, anesthesiologists ensure patient comfort and safety during sedation or anesthesia.

Pharmacists: Medication management is a critical aspect of arrhythmia care. Pharmacists can assist in selecting the right medications, adjusting dosages, and monitoring for potential drug interactions [40].

Benefits of a Multidisciplinary Approach

Individualized care: Each patient's arrhythmia journey is unique. A multidisciplinary team considers the patient's specific condition, medical history, preferences, and goals to develop a personalized treatment plan [41].

Comprehensive evaluation: Patients benefit from a comprehensive evaluation that includes a range of perspectives and specialties. This ensures that all aspects of their health are considered in the treatment plan [42].

Optimized treatment: Collaboration fosters an environment where specialists can discuss and refine treatment strategies. This can lead to more effective and less invasive interventions [43].

Enhanced communication: Multidisciplinary teams facilitate communication among healthcare professionals, reducing the risk of errors, missed diagnoses, or mismanagement [44,45].

## Conclusions

In conclusion, holistic arrhythmia management represents the future of patient-centered cardiology. Collaboration between healthcare professionals, such as cardiologists, electrophysiologists, nurses, and pharmacists, highlights the value of a multidisciplinary approach. This synergy promotes thorough comprehension and individualized treatment plans. The holistic management of arrhythmias integrates multiple therapies. Combining medications, catheter ablation, and devices permits a multifaceted approach, which improves treatment success. Medication provides immediate alleviation, ablation offers the possibility of cures, and devices provide protection. Individualized care is essential in the management of arrhythmias. Personalized treatment plans aligned with each patient's specific requirements increase patient satisfaction and adherence while minimizing unnecessary interventions. Expect enhanced medications, ablation techniques, and devices as arrhythmia management advances. Precise, side-effect-reduced drugs will empower patients. Enhanced ablation instruments will improve the safety and efficacy of procedures. Smaller, longer-lasting devices will improve portability. These developments provide patients and clinicians with optimism. Patients with arrhythmias can anticipate a brighter future due to the availability of less invasive treatment options. Clinicians can refine their methods to provide optimal care. In essence, the future of arrhythmia management is bright. Holistic care, which emphasizes collaboration and individualization, reflects the evolution of medicine and affords those struggling with these conditions a better quality of life.
